# Pelvic pain symptoms and endometriosis characteristics in relation to oxidative stress among adolescents and adults with and without surgically-confirmed endometriosis

**DOI:** 10.12688/f1000research.141793.1

**Published:** 2024-01-08

**Authors:** Amy L Shafrir, Britani Wallace, Ashley Laliberte, Allison F Vitonis, Christine B Sieberg, Kathryn L Terry, Stacey A Missmer

**Affiliations:** 1 Boston Center for Endometriosis, Boston Children's Hospital and Brigham and Women's Hospital, Boston, Massachusetts, 02115, USA; 2 Division of Adolescent and Young Adult Medicine, Department of Pediatrics, Boston Children's Hospital and Harvard Medical School, Boston, MA, 02115, USA; 3 Department of Obstetrics, Gynecology, and Reproductive Biology, Brigham and Women's Hospital and Harvard Medical School, Boston, MA, 02115, USA; 4 Department of Pyschiatry, Harvard Medical School, Boston, MA, 02115, USA; 5 Pain & Affective Neuroscience Center, Department of Anesthesiology, Boston Children's Hospital, Boston, MA, 02115, USA; 6 Biobehavioral Pain Innovations Lab, Department of Psychiatry & Behavioral Sciences, Boston Children's Hospital, Boston, MA, 02115, USA; 7 Department of Epidemiology, Harvard T.H. Chan School of Public Health, Boston, MA, 02115, USA; 8 Department of Obstetrics, Gynecology, and Reproductive Biology, College of Human Medicine, Michigan State University, Grand Rapids, MI, 49503, USA

**Keywords:** oxidative stress, dysmenorrhea, pelvic pain, dyspareunia, endometriosis, endometriotic lesions

## Abstract

**Background:** While the majority of reproductive-aged females will experience pelvic pain during their lives, biological mechanisms underlying pelvic pain are not well understood. We investigated associations between pelvic pain symptoms and oxidative stress among people with and without surgically-confirmed endometriosis.

**Methods:** Using an enzyme-linked immunosorbent assay, we measured 8-Hydroxy-2’-deoxyguanosine (8-OHdG) in urine samples and corrected for creatinine levels in 434 surgically-confirmed endometriosis participants compared to 605 participants never diagnosed with endometriosis. At enrollment, participants reported details of their pelvic pain symptoms. Linear regression was used to compute geometric mean (GM) creatinine-corrected 8-OHdG levels with 95% confidence intervals (CI) among all participants and those with and without endometriosis separately, adjusting for potential confounders. Interactions by surgically-confirmed endometriosis status were tested by Wald statistics.

**Results:** No trends in 8-OHdG were observed among those with or without endometriosis for severity or frequency of dysmenorrhea, acyclic pelvic pain, dyspareunia or pain with bowel movements. Among endometriosis participants, lower 8-OHdG levels were observed for participants with any white, blue/black, or brown lesions (GM=76.7 versus 82.9 ng/mg; p=0.10), which was primarily driven by lower levels of 8-OHdG for any blue/black lesions (GM=72.8 versus 81.6 ng/mg; p=0.05).

**Conclusion:** While no associations were observed between 8-OHdG and pelvic pain symptoms, future research is needed to assess how other pathways of oxidative damage, e.g. through proteins or lipids, may affect endometriosis-associated symptoms. Additionally, further research is needed to understand differences in oxidative stress among endometriosis lesion sub-phenotypes.

## Introduction

The vast majority of reproductive-aged females will experience pelvic pain at some point in their lives. Upwards of 90% of females report experiencing dysmenorrhea ^1^ and 15-20% report chronic pelvic pain. ^2^ Pelvic pain can result in reduced quality of life, decreased work productivity, and substantial healthcare costs. While for some individuals, morbidity that may be causing their pelvic pain will be found, (e.g., endometriosis, uterine fibroids), others will struggle to find a gynecologic pathology that may explain their pain. At least 30% of individuals undergoing a laparoscopic surgery for chronic pelvic pain will have no visualized pathology. ^3^ Among those with endometriosis, lesion location and revised American Society for Reproductive Medicine (rASRM) staging have not correlated with pelvic pain severity or pain remediation. ^4^^–^^6^ Understanding the biological mechanisms underlying pelvic pain could help to advance treatment options to alleviate these life-impacting symptoms.

While inflammatory pathways have been implicated in pelvic pain, ^7^ less is known about the involvement of oxidative stress on pelvic pain symptoms. Normal cellular processes lead to the production of reactive oxygen species (ROS) that can cause tissue damage most notably to proteins, lipids, and DNA. Counteracting antioxidant mechanisms, such as neutralizing ROS, help to reduce the number of ROS in cells. Oxidative stress occurs when the balance between ROS and antioxidants begins to shift, due to either a decrease of antioxidant products or an increase in ROS. Higher levels of oxidative stress have been shown to be present in individuals with fibromyalgia, characterized by widespread pain, and to increase with increasing fibromyalgia pain severity. ^8^ Additionally, oxidative stress is one of the factors implicated in the development and progression of diabetic peripheral neuropathy. ^9^ Limited studies have assessed the association between pelvic pain symptoms and oxidative stress, with the majority observing an increase in oxidative stress and dysmenorrhea (period pain). ^10^^–^^15^ However, most of the studies did not adjust for important potential confounders, such as age, body mass index, and cigarette smoking status, which affect oxidative stress levels, and did not assess associations between oxidative stress and pelvic pain symptoms other than dysmenorrhea.

Further, one of the challenges of studying oxidative stress is that oxidative products are difficult to measure. Free radicals have a very short half-life and thus markers of oxidative damage are often utilized to measure the extent of oxidative stress within an individual. While blood contains organic and inorganic metal content, which can be oxidized during sample collection and storage, urine contains far less organic and inorganic metal content and as such is less likely to have misclassification of *in vivo* oxidative stress levels due to collection and storage handling. ^16^ 8-Hydroxy-2′-deoxyguanosine (8-OHdG) also known as 8-oxo-7,8-dihydro-2′-deoxyguanosine (8-oxodG) is a widely used marker of DNA oxidative stress. ^16^ After DNA repair due to oxidative stress, 8-OHdG is excreted in urine and has been shown to be a reliable marker of oxidative stress. ^17^ Previous studies have noted associations between 8-OHdG and cancer, atherosclerosis, and diabetes pathogenesis. ^17^ However, only one study has assessed pelvic pain symptoms and urinary 8-OHdG levels. This recent study among 188 female university students found no statistical association between dysmenorrhea and 8-OHdG levels. ^13^

Therefore, we sought to understand how oxidative stress measured by urinary 8-OHdG may be related to pelvic pain symptoms. In addition, we explored if these associations may be unique to individuals with endometriosis, a condition commonly found among those with chronic pelvic pain, or play a broader role in pelvic pain symptoms. We investigated the association between dysmenorrhea, acyclic pelvic pain and dyspareunia presence, severity and frequency, as well as pain with bowel movements, in relation to urinary 8-OHdG levels among females with and without surgically-confirmed endometriosis. Additionally, among those with endometriosis, we investigated the association between surgically visualized endometriotic lesion characteristics and urinary 8-OHdG levels.

## Methods

The Women’s Health Study: From Adolescence to Adulthood (A2A) cohort enrolled adolescents and adults oversampled for those surgically diagnosed with endometriosis from 2012 to 2018. ^18^^,^^19^ Those with endometriosis (n=785) were enrolled from Brigham and Women’s Hospital (BWH) and Boston Children’s Hospital (BCH) and were eligible if they were 1) female; 2) aged 7-55 years; and 3) had a surgical diagnosis of endometriosis. Population and clinic sampled participants without any diagnosis of endometriosis (n=764) were recruited from the local Boston community through local advertisements, online postings, and word of mouth and from BWH and BCH clinics. These participants were eligible if they were females aged 7-55 years without any diagnosis of endometriosis. Those never diagnosed with endometriosis are referred to as “participants without endometriosis” in the manuscript. The study was approved by the BCH Institutional Review Board on behalf of both BCH and BWH (Approval number: P00004267; Approval date: 09/11/2012). Written informed consent was obtained from participants with both parental consent and participant assent for participants less than 18 years of age at enrollment.

At enrollment, participants completed an extensive baseline questionnaire to assess behavioral and reproductive factors, pain symptoms, quality of life, and medication use that expands upon the World Endometriosis Research Foundation (WERF) Endometriosis Phenome and Biobanking Harmonization Project (EPHect) standard clinical questionnaire. ^20^ Survey data was managed with REDCap electronic data capture tools. ^21^

### Pain symptom assessment

Detailed information was collected on the baseline questionnaire on the presence, severity, and frequency of dysmenorrhea (pain with periods), acyclic/general pelvic pain (pain not associated with menses), and dyspareunia (pain with sexual vaginal intercourse/penetration). Usual severity of dysmenorrhea was assessed categorically as none, mild (medication never or rarely needed), moderate (medication usually needed), and severe (medication and bed rest needed). Dysmenorrhea frequency within the past 12 months was assessed as never, occasionally, often, usually, and always. For acyclic pelvic pain, participants reported if they had experienced acyclic pelvic pain within the past three months. Among those with acyclic pelvic pain in the past three months, the 11-point numeric rating scale (NRS) was used to assess the acyclic pelvic pain severity during that timeframe, with 0=no pain and 10=worst pain imaginable. Acyclic pelvic pain frequency was assessed as less than monthly, monthly, weekly, and daily. Among participants aged 18 or older, participants reported if they had experienced dyspareunia in the last 12 months. Among those reporting dyspareunia, the 11-point NRS scale was used to assess severity while frequency of dyspareunia during or in the 24 hours after intercourse/penetration in the past 12 months was assessed as never, occasionally, often, usually, and always. Finally, participants reported if they had pain with bowel movements in the past 12 months. Those who reported pain rated the severity of their pain on the 11-point NRS scale.

### Endometriotic lesion characteristics

The WERF EPHect surgical form was used to capture information on rASRM score, endometriosis subtype, and endometriosis lesion(s) color and location at the surgery closest to urine collection for endometriosis cases. ^22^ We further categorized endometriosis lesions into colors that are normally observed earlier in the lesion progression (i.e. red, yellow, and clear lesions) and later in the lesion progression (i.e. blue, black/brown, and white lesions).

### Urine collection

Urine samples were collected at baseline in compliance with the WERF EPHect standardized fluids tools, ^23^ with the exception that we did not require clean catch collection of urine samples. Participants completed a biospecimen questionnaire at the time of sample collection on which they reported date of last menstrual period, timing of last foods/beverages consumed, and recent medication and hormone use. All urine samples were aliquoted into cryovials and stored at -80 ^o^C until assayed.

### Oxidative stress measurement

8-OHdG was measured in urine using the HT 8-oxo-dG ELISA Kit II (R&D Systems, Inc. Minneapolis, MN, USA) at the Clinical and Epidemiology Laboratory at Boston Children’s Hospital (BCH). Creatinine levels were also measured in the urine samples using an FDA-approved enzymatic assay method on the Roche Cobas 6000 system using Roche Diagnostics reagents (Indianapolis, IN) at the Clinical and Epidemiology Laboratory at BCH. Approximately 2-3 blinded quality control (QC) urine samples were distributed randomly within each batch. The coefficient of variation (CV) in blinded QC samples for 8-OHdG was 13% and for creatinine was 1.5%.

### Covariates

Information on covariates was collected on the baseline questionnaire and biospecimen form and included: age at urine collection (continuous), cigarette smoking history (never, former, current), age at menarche (continuous), menstrual cycle phase at time of urine collection (follicular, peri-ovulatory, luteal), hormonal medication use within 30 days from urine collection (yes, no), and pain medication use within 48 hours from urine collection (yes, no). Additionally, participants reported their menstrual period frequency in the past 12 months. We calculated body mass index (BMI) as kg/m ^2^ based on self-reported weight and height. For women aged ≥20 years, BMI was categorized according to the World Health Organization Criteria: underweight (BMI < 18.5 kg/m ^2^), normal weight (18.5-24.9 kg/m ^2^), overweight (25-29.9 kg/m ^2^), and obese (≥30 kg/m ^2^). For those <20 years, the age- and sex-specific BMI Z-score was calculated and participants were categorized as underweight (Z-score ≤-2), normal weight (Z-score >-2 to <1), overweight (Z-score 1-2), and obese (Z-score >2). Participants were also asked to report their physical activity levels at baseline. Participants reported the average time per week they spent doing various activities (e.g. walking/hiking, jogging, running, lap swimming, playing various sports) in the past year. The 2011 Compendium of Physical Activities was then used to assign metabolic equivalent hours (MET-hours) per week to each of the activities. ^24^ We multiplied the reported hours per week engaged in each activity by the appropriate MET score for that activity (e.g. 4 for walking, 8.3 for bicycling, 11.7 for running) and summed the values for the individual activities to create MET-h/wk of total activity. For those with surgically-confirmed endometriosis, information on age at first endometriosis symptoms, number of physicians seen until diagnosed, and time between first symptoms and surgical diagnosis also were reported on the baseline questionnaire.

Also at baseline, participants completed a semi-quantitative Food Frequency Questionnaire (FFQ), which included over 130 items on the consumption of a range of foods and beverages. The FFQ was utilized to calculate the Alternative Healthy Eating Index (AHEI) score for all participants. Details on this scoring method can be found elsewhere. ^25^^,^^26^ Briefly, AHEI scoring is based on the consumption of fruit, vegetables, whole grains, sugar-sweetened beverages and fruit juices, nuts and legumes, red/processed meat, trans fat, long-chain (n-3) fats (EPA + DHA), polyunsaturated fat, sodium, and alcohol. Given the younger age of this cohort (37% <21 years old at baseline), we omitted alcohol from the AHEI calculation. The AHEI score is summed across all of the components and ranges from 0 to 100, with higher scores representing dietary patterns that are more aligned with healthy eating.

### Statistical analysis

Of the 1549 participants enrolled in the A2A cohort, 1209 provided a urine sample at baseline and all 549 endometriosis participants and 660 participants without endometriosis had 8-OHdG and creatinine measured in their baseline urine sample. Of these 1209 participants, we excluded participants who did not complete the questionnaire at baseline (5 endometriosis, 3 without endometriosis) or completed the questionnaire at baseline more than 60 days before/after their urine collection (105 endometriosis, 36 without endometriosis). We also excluded incident endometriosis participants (3 participants without endometriosis at enrollment diagnosed with endometriosis up to 3 years after enrollment) and those who were premenarchal or never cycled (5 endometriosis, 12 without endometriosis) for a final analytic sample size of 434 surgically diagnosed endometriosis participants and 606 participants without endometriosis. Dysmenorrhea analyses were restricted to participants who reported having menstrual periods in the past three months (264 endometriosis and 517 without endometriosis). Analyses of acyclic pelvic pain severity and frequency were restricted to participants who reported having acyclic pelvic pain in the past 3 months (268 endometriosis and 78 without endometriosis). Additionally, analyses of dyspareunia severity and frequency were restricted to participants age ≥18 who reported experiencing dyspareunia in the past 12 months (132 endometriosis and 136 without endometriosis). Analyses of endometriotic lesion characteristics were restricted to the 380 participants with a WERF EPHect surgical form completed at their most recent endometriosis surgery.

8-OHdG and creatinine levels were log-transformed to improve normality. To adjust for differences in urine volume, we divided the 8-OHdG measurement by the creatinine measurement to calculate creatinine-corrected 8-OHdG levels (ng/mg). We then used the generalized extreme studentized deviate many-outlier detection approach to identify statistical outliers for creatinine-corrected 8-OHdG. ^27^ For batch adjustment, levels of creatinine-corrected 8-OHdG were recalibrated to have a comparable distribution to an average batch according to the methods described by Rosner and colleagues. ^28^

After exclusions, we used linear regression to calculate geometric means (GM) and 95% confidence intervals (CI) for creatinine-corrected 8-OHdG levels, adjusting for age (continuous), hormone use within the 30 days prior to urine collection (yes, no), any pain medication use within 48 hours prior to urine collection (yes, no), Alternative Healthy Eating Index (quartiles), body mass index (underweight, normal weight, overweight, obese), and physical activity (quartiles of MET-hrs/week). Analyses were conducted among all participants and separately among those with and without endometriosis. Participants were excluded from analyses if they were missing the main pain symptom exposure variable. We calculated trend tests by modeling the categorical pain exposure variable as ordinal, adjusting for the same variables listed above. Pairwise comparisons between levels of categorical variables with three categories or more were performed using the Tukey adjustment for multiple testing. To evaluate if associations between pain symptoms and 8-OHdG levels differed between participants with and without endometriosis, we included an interaction term for endometriosis diagnosis status (endometriosis vs. no endometriosis) and each pain symptom in the linear regression models. The Wald statistic was used to calculate the two-sided p-value for interaction.

In sensitivity analyses, we restricted analyses to never smokers as cigarette smoking is known to have a significant effect on oxidative stress levels. Additionally, we excluded endometriosis participants who had an endometriosis-related surgery before they completed their baseline questionnaire and/or their baseline urine collection to remove the affects surgery may have had on oxidative stress levels and pain symptoms. All statistical analyses were performed using SAS version 9.4 (SAS Institute Inc., Cary, NC) and all p-values are two-sided.

## Results

### Study population characteristics

We included 434 surgically confirmed endometriosis participants and 606 participants without endometriosis in our analyses. On average, baseline questionnaires were completed 6.8 days (standard deviation=17.5) prior to urine collection. At enrollment, those with endometriosis were younger (median age 18 vs. 24 years), and a higher proportion were overweight (25% vs. 20%) and identify as White race (91% vs. 71%) compared to those never diagnosed with endometriosis ( Table 1). Additionally, participants without endometriosis were more likely to have had a period in the last three months (85% vs. 61%) compared to endometriosis participants, while endometriosis participants were more likely to have taken hormonal medications within 30 days of urine collection (87% vs. 54%) and pain medication within 48 hours of urine collection (23% vs. 18%) compared to those without endometriosis. Participants without endometriosis also reported higher physical activity and AHEI scores compared to endometriosis participants.

**Table 1.** Characteristics and creatinine-corrected 8-OHdG levels for A2A participants with and without surgically-diagnosed endometriosis ^1^.

	Endometriosis	No endometriosis ^2^
	(N=434)	(N=606)
Creatinine-corrected 8-OHdG (ng/mg) ^3^		
Geometric Mean (95% CI)	82.5 (78.5, 86.7)	80.4 (77.2, 83.8)
Age at urine collection (years)		
Median (IQR)	18 (16-23)	24 (22-28)
Race		
Black	10 (2%)	41 (7%)
White	393 (91%)	433 (71%)
Other/Unknown ^4^	31 (7%)	132 (22%)
Ethnicity		
Hispanic	32 (7%)	54 (9%)
Non-Hispanic	402 (93%)	552 (91%)
Body mass index (kg/m ^2^) ^5^		
Underweight	7 (2%)	24 (4%)
Normal weight	259 (60%)	401 (66%)
Overweight	110 (25%)	122 (20%)
Obese	58 (13%)	58 (10%)
Cigarette smoking status		
Never	391 (95%)	552 (94%)
Ever	19 (5%)	38 (6%)
Age at menarche (years)		
Median (IQR)	12 (11-13)	12 (11-13)
Self-reported periods in the past 3 months ^6^		
No	166 (39%)	89 (15%)
Yes	264 (61%)	517 (85%)
Menstrual cycle phase at urine collection ^7^		
Follicular	8 (42%)	73 (50%)
Peri-ovulation	3 (16%)	17 (12%)
Luteal	8 (42%)	57 (39%)
Hormonal medication use within 30 days prior to urine collection		
Not taking hormones	53 (13%)	276 (46%)
Taking hormones	365 (87%)	325 (54%)
Pain medication used within 48 hours prior to urine collection		
No	333 (77%)	499 (82%)
Yes	101 (23%)	107 (18%)
Physical activity (met-hours/week)		
Median (IQR)	31.6 (10.9-65.5)	36.5 (19.6-70.4)
Alternative Healthy Eating Index		
Median (IQR)	51.6 (43.8-59.9)	61.4 (53.0-71.7)

^1^ Categories do not all add up to 434 participants with endometriosis and 606 without endometriosis due to missing values (BMI: no endometriosis=1; smoking: endometriosis=24, no endometriosis=16; age at menarche: no endometriosis=4; period in last 3 months: endometriosis=4; menstrual cycle phase: endometriosis=5, no endometriosis=8; hormone use: endometriosis=16, no endometriosis=5; physical activity: endometriosis=46, no endometriosis=77; Alternative Healthy Eating Index: endometriosis=51, no endometriosis=86).

^2^ Participants without endometriosis did not have a surgical diagnosis of endometriosis.

^3^ Geometric mean levels adjusted for age (continuous), hormone use within the 30 days prior to urine collection (yes, no), pain medication use within 48 hours prior to urine collection (yes, no), Alternative Healthy Eating Index (quartiles), body mass index (underweight, normal weight, overweight, obese), and physical activity (quartiles of MET-hrs/week).

^4^ Participants in the Other/Unknown category included American Indian/Alaska Native (endometriosis: 1, no endometriosis: 0), Asian (endometriosis: 2, no endometriosis: 84), Native Hawaiian or Pacific Islander (endometriosis: 0, no endometriosis: 1), Multiracial (endometriosis: 13, no endometriosis: 32), other race (endometriosis: 14, no endometriosis: 11), unknown (endometriosis: 1, no endometriosis: 4).

^5^ For women aged ≥20 years: underweight (BMI < 18.5 kg/m ^2^), normal weight (BMI 18.5–24.9 kg/m ^2^), overweight (BMI 25–29.9 kg/m ^2^), or obese (BMI ≥ 30 kg/m ^2^) according to World Health Organization criteria; For those <20 years, the age- and gender-specific BMI Z-score was calculated, and participants were categorized as underweight (Z-score ≤−2), normal weight (Z-score >−2 to <1), overweight (Z-score 1–2), or obese (Z-score >2).

^6^ Due to the phrasing in the questionnaire, participants who self-reported having periods could have been on cyclic hormone therapy or having bleeding despite being on continuous hormones.

^7^ Among participants with self-reported menstrual periods in the past 3 months, who were not on hormones and whose menstrual cycles were not long or irregular.

### Pelvic pain symptoms

For pelvic pain symptoms, we observed no associations between 8-OHdG and dysmenorrhea (severity, frequency), acyclic pelvic pain (presence, frequency, or severity), dyspareunia (presence, frequency, or severity) as well as severity of pain with bowel movements ( Table 2). Results between pain symptoms and 8-OHdG were similar when restricted to never cigarette smokers ( Table 3). Additionally, results were similar when restricted to participants with urine collection and questionnaire completion before their baseline surgery ( Table 4).

**Table 2.** Presence, severity, and frequency of dysmenorrhea, acyclic pelvic pain, and dyspareunia as well as severity of pain with bowel movements in relation to creatinine-corrected 8-OHdG levels among A2A participants (N=1039) ^1^.

	All participants		Endometriosis		No endometriosis	
		Creatinine-corrected 8-OHdG (ng/mg)		Creatinine-corrected 8-OHdG (ng/mg)		Creatinine-corrected 8-OHdG (ng/mg)
	N	Geometric mean (95% CI)	N	Geometric mean (95% CI)	N	Geometric mean (95% CI)
**Dysmenorrhea** ^**2**^						
Severity ^3^						
None/Mild	347 (45%)	80.1 (76.1, 84.4)	15 (6%)	81.0 (64.5, 101.6)	332 (65%)	81.6 (77.4, 86.1)
Moderate	213 (28%)	85.3 (80.0, 91.0)	80 (31%)	87.6 (79.3, 96.7)	133 (26%)	82.8 (76.1, 90.1)
Severe	211 (27%)	80.0 (74.8, 85.6)	167 (64%)	79.2 (74.0, 84.8)	44 (9%)	75.2 (64.9, 87.2)
*p-value* *^4^*		0.26		0.28		0.53
*p-interaction* *^5^*						0.90
Frequency ^6^ ^,^ ^7^						
Never/Rarely/Occasionally	311 (50%)	79.1 (74.8, 83.6)	17 (9%)	92.1 (73.4, 115.7)	294 (67%)	79.9 (75.5, 84.6)
Often/Usually	81 (13%)	84.7 (76.1, 94.2)	18 (10%)	81.8 (65.5, 102.2)	63 (14%)	85.7 (75.7, 97.0)
Always	227 (37%)	83.0 (77.7, 88.6)	144 (80%)	79.2 (73.3, 85.6)	83 (19%)	84.1 (75.5, 93.7)
*p-trend* *^8^*		0.27		0.24		0.32
*p-interaction* *^5^*						0.20
**Acyclic pelvic pain**						
Presence last 3 months ^9^						
No	666 (66%)	81.9 (78.9, 84.9)	153 (36%)	80.3 (74.8, 86.2)	513 (87%)	83.6 (80.1, 87.2)
Yes	346 (34%)	81.9 (77.8, 86.2)	268 (64%)	78.9 (74.8, 83.2)	78 (13%)	84.7 (75.9, 94.6)
*p-value*		0.98		0.71		0.82
*p-interaction* *^5^*						0.58
Severity ^10^ ^,^ ^11^						
Mild	52 (16%)	81.9 (72.4, 92.8)	27 (10%)	83.0 (70.7, 97.5)	25 (35%)	83.0 (66.9, 102.8)
Moderate	75 (23%)	80.8 (72.9, 89.7)	52 (20%)	78.7 (70.0, 88.4)	23 (32%)	85.8 (68.6, 107.4)
Severe	203 (61%)	80.3 (75.4, 85.5)	180 (69%)	80.0 (75.2, 85.2)	23 (32%)	79.9 (63.8, 100.0)
*p-trend* *^8^*		0.78		0.82		0.82
*p-interaction* *^5^*						0.80
Frequency ^10^ ^,^ ^12^						
Monthly or less	144 (43%)	82.4 (76.3, 89.0)	87 (33%)	82.5 (75.2, 90.5)	57 (74%)	86.0 (74.5, 99.3)
Weekly or daily	193 (57%)	78.3 (73.2, 83.6)	173 (67%)	77.4 (72.5, 82.6)	20 (26%)	75.9 (59.1, 97.5)
*p-value*		0.33		0.28		0.57
*p-interaction* *^5^*						0.59
**Dyspareunia** ^**13**^						
Presence in last 12 months						
No	339 (56%)	80.8 (76.7, 85.0)	35 (20%)	73.6 (64.1, 84.4)	304 (67%)	82.8 (78.3, 87.6)
Yes	268 (44%)	83.7 (79.0, 88.6)	132 (76%)	77.6 (72.4, 83.2)	136 (30%)	87.1 (80.0, 94.7)
*p-value*		0.38		0.50		0.34
*p-interaction* *^5^*						0.99
Severity ^14^ ^,^ ^15^						
Mild	85 (35%)	80.1 (72.7, 88.3)	28 (22%)	75.9 (65.1, 88.6)	57 (42%)	84.7 (74.1, 96.8)
Moderate	83 (34%)	85.2 (77.1, 94.1)	30 (23%)	79.4 (68.3, 92.2)	53 (39%)	89.4 (77.8, 102.6)
Severe	97 (40%)	79.6 (72.7, 87.3)	72 (55%)	75.7 (68.8, 83.3)	25 (19%)	84.9 (69.4, 103.9)
*p-trend* *^8^*		0.90		0.89		0.87
*p-interaction* *^5^*						0.76
Frequency ^14^						
Occasionally	123 (46%)	80.0 (73.8, 86.7)	33 (25%)	74.4 (64.8, 85.6)	90 (66%)	83.8 (75.6, 92.9)
Often/Usually	98 (37%)	85.3 (77.9, 93.3)	59 (45%)	78.2 (70.5, 86.9)	39 (29%)	95.8 (81.9, 112.0)
Always	47 (17%)	78.4 (68.8, 89.3)	40 (30%)	76.7 (67.6, 87.2)	7 (5%)	73.2 (50.3, 106.7)
*p-trend* *^8^*		0.92		0.77		0.61
*p-interaction* *^5^*						0.78
**Pain with bowel movements**						
Severity ^16^ ^,^ ^17^						
None/Mild	768 (78%)	82.2 (79.5, 85.1)	249 (62%)	80.6 (76.2, 85.3)	519 (88%)	83.3 (79.9, 87.0)
Moderate	136 (14%)	80.9 (74.6, 87.7)	85 (21%)	77.5 (70.4, 85.3)	51 (9%)	84.0 (73.3, 96.3)
Severe	86 (9%)	81.2 (73.3, 90.0)	68 (17%)	79.3 (71.1, 88.4)	18 (3%)	86.3 (68.6, 108.8)
*p-trend* *^8^*		0.73		0.65		0.77
*p-interaction* *^5^*						0.43

^1^ All p-values are two-sided and were adjusted for age (continuous in years), hormone use within the prior 30 days of urine collection (yes, no), pain medication use within the prior 48 hours of urine collection (yes, no), Alternative Healthy Eating Index (quartiles), body mass index (underweight, normal weight, overweight, obese), and physical activity (quartiles of MET-hrs/week). P-values for interactions between endometriosis and comparison participants were calculated using the Wald statistic.

^2^ Restricted to 264 endometriosis and 517 no endometriosis participants who reported having periods in the last 3 months. Due to the phrasing in the questionnaire, participants who reported having periods could have been on cyclic hormone therapy or having bleeding despite being on continuous hormones.

^3^ Missing dysmenorrhea severity: 2 endometriosis and 8 no endometriosis participants.

^4^ P-value for any difference between three dysmenorrhea severity groups.

^5^ P-value for test of interaction for those with and without endometriosis.

^6^ Restricted to participants who answered the baseline questionnaire from January 2014 onwards when this question was added and to participants who reported having periods in the last 3 months (183 endometriosis and 452 no endometriosis participants).

^7^ Missing dysmenorrhea frequency: 4 endometriosis and 12 no endometriosis participants.

^8^ P-value for linear test for trend modeling the exposure as ordinal.

^9^ Missing presence of acyclic pelvic pain: 13 endometriosis and 15 no endometriosis participants.

^10^ Among participants who reported acyclic pelvic pain in the past three months.

^11^ Missing acyclic pelvic pain severity: 9 endometriosis and 7 no endometriosis participants.

^12^ Missing acyclic pelvic pain frequency: 8 endometriosis and 1 no endometriosis participants.

^13^ Among participants who had intercourse in the past 12 months (excluded 195 endometriosis and 17 no endometriosis aged <18 years, 3 endometriosis and 36 no endometriosis who declined to be asked dyspareunia questions, 57 endometriosis and 85 no endometriosis who had never had intercourse, 7 endometriosis and 14 no endometriosis had not had intercourse in the past 12 months, 5 endometriosis and 14 no endometriosis were missing information on dyspareunia).

^14^ Among participants age ≥18 with dyspareunia in past 12 months.

^15^ Missing dyspareunia severity: 2 endometriosis and 1 no endometriosis participants.

^16^ Based on 0-10 numeric rating scale categorized as none/mild (0-3), moderate (4-6), severe (7-10).

^17^ Missing severity of pain with bowel movements: 32 endometriosis and 18 no endometriosis participants.

**Table 3.** Presence, severity, and frequency of dysmenorrhea, acyclic pelvic pain, and dyspareunia as well as severity of pain with bowel movements in relation to 8-OHdG levels restricted to A2A participants who had never smoked (N=942) ^1^.

	All participants		Endometriosis		No endometriosis	
		Creatinine-corrected 8-OHdG (ng/mg)		Creatinine-corrected 8-OHdG (ng/mg)		Creatinine-corrected 8-OHdG (ng/mg)
	N=942	Geometric mean (95% CI)	N=391	Geometric mean (95% CI)	N=552	Geometric mean (95% CI)
**Dysmenorrhea** ^**2**^						
Severity						
None/Mild	328 (46%)	79.8 (75.6, 84.2)	13 (6%)	85.9 (67.1, 110.0)	315 (67%)	81.0 (76.7, 85.6)
Moderate	194 (27%)	85.0 (79.4, 91.1)	75 (32%)	86.6 (77.9, 96.1)	119 (25%)	82.3 (75.3, 90.1)
Severe	185 (26%)	79.8 (74.2, 85.8)	146 (62%)	79.5 (73.8, 85.7)	39 (8%)	74.4 (63.5, 87.1)
*p-value* *^3^*		0.30		0.42		0.54
*p-interaction* *^4^*						0.99
Frequency ^5^ ^,^ ^6^						
Never/Rarely/Occasionally	292 (51%)	78.7 (74.3, 83.5)	17 (11%)	91.7 (72.9, 115.4)	275 (67%)	79.4 (74.8, 84.2)
Often/Usually	75 (13%)	84.6 (75.7, 94.7)	17 (11%)	79.5 (63.0, 100.3)	58 (14%)	86.0 (75.5, 98.0)
Always	205 (36%)	81.9 (76.3, 87.8)	127 (79%)	78.4 (72.1, 85.2)	78 (19%)	82.9 (74.0, 92.7)
*p-trend* *^7^*		0.38		0.25		0.39
*p-interaction* *^4^*						0.21
**Acyclic pelvic pain**						
Presence last 3 months ^8^						
No	620 (66%)	80.9 (77.8, 84.0)	143 (37%)	79.8 (74.1, 86.0)	477 (87%)	82.3 (78.7, 86.1)
Yes	314 (34%)	82.1 (77.8, 86.7)	243 (63%)	79.0 (74.6, 83.6)	71 (13%)	85.5 (76.1, 96.0)
*p-value*		0.66		0.82		0.56
*p-interaction* *^4^*						0.41
Severity ^9^ ^,^ ^10^						
Mild	48 (16%)	82.5 (72.3, 94.2)	24 (10%)	82.7 (69.6, 98.3)	24 (37%)	83.9 (66.8, 105.5)
Moderate	69 (23%)	80.4 (72.0, 89.7)	48 (21%)	78.6 (69.5, 88.9)	21 (32%)	84.9 (66.5, 108.4)
Severe	182 (61%)	80.2 (75.0, 85.9)	162 (69%)	80.3 (75.1, 85.8)	20 (31%)	77.9 (60.6, 100.3)
*p-trend* *^7^*		0.74		0.90		0.69
*p-interaction* *^4^*						0.65
Frequency ^9^ ^,^ ^11^						
Monthly or less	133 (44%)	82.9 (76.4, 89.9)	81 (34%)	82.4 (74.7, 90.8)	52 (74%)	87.0 (74.5, 101.6)
Weekly or daily	172 (56%)	78.0 (72.6, 83.8)	154 (66%)	77.4 (72.1, 83.0)	18 (26%)	74.7 (56.9, 98.2)
*p-value*		0.28		0.31		0.36
*p-interaction* *^4^*						0.70
**Dyspareunia** ^**12**^						
Presence in last 12 months						
No	315 (57%)	80.4 (76.2, 84.8)	31 (22%)	76.1 (65.4, 88.4)	284 (70%)	82.1 (77.4, 87.0)
Yes	233 (43%)	82.7 (77.7, 88.0)	110 (78%)	76.7 (70.9, 83.0)	123 (30%)	85.5 (78.2, 93.5)
*p-value*		0.51		0.92		0.45
*p-interaction* *^4^*						0.77
Severity ^13^ ^,^ ^14^						
Mild	75 (32%)	79.7 (71.7, 88.7)	22 (20%)	78.9 (66.0, 94.3)	53 (43%)	82.2 (71.4, 94.8)
Moderate	75 (32%)	84.7 (76.0, 94.2)	27 (25%)	77.6 (65.7, 91.5)	48 (39%)	89.0 (76.8, 103.2)
Severe	81 (36%)	77.6 (70.1, 86.0)	60 (55%)	73.8 (66.3, 82.2)	21 (17%)	83.5 (66.8, 104.4)
*p-trend* *^7^*		0.71		0.50		0.76
*p-interaction* *^4^*						0.43
Frequency ^14^						
Occasionally	111 (48%)	79.3 (72.7, 86.5)	27 (25%)	72.7 (61.9, 85.4)	84 (68%)	83.1 (74.5, 92.7)
Often/Usually	82 (35%)	82.5 (74.6, 91.4)	49 (45%)	76.1 (67.5, 85.9)	33 (27%)	92.6 (77.8, 110.2)
Always	40 (17%)	80.3 (69.4, 92.9)	34 (31%)	78.1 (67.7, 90.1)	6 (5%)	73.8 (48.7, 112.0)
*p-trend* *^7^*		0.76		0.51		0.72
*p-interaction* *^4^*						0.94
**Pain with bowel movements**						
Severity ^15^ ^,^ ^16^						
None/Mild	716 (78%)	81.7 (78.8, 84.7)	234 (63%)	80.7 (76.1, 85.6)	482 (88%)	82.5 (78.8, 86.2)
Moderate	126 (14%)	80.7 (74.1, 87.8)	79 (21%)	77.8 (70.3, 86.0)	47 (9%)	83.7 (72.6, 96.6)
Severe	78 (8%)	80.0 (71.8, 89.2)	61 (16%)	77.9 (69.5, 87.4)	17 (3%)	85.3 (67.1, 108.5)
*p-trend* *^7^*		0.67		0.51		0.74
*p-interaction* *^4^*						0.36

^1^ All p-values are two-sided and were adjusted for age (continuous in years), hormone use within the prior 30 days of urine collection (yes, no), pain medication use within the prior 48 hours of urine collection (yes, no), Alternative Healthy Eating Index (quartiles), body mass index (underweight, normal weight, overweight, obese), and physical activity (quartiles of MET-hrs/week). P-values for interactions between endometriosis and comparison participants were calculated using the Wald statistic.

^2^ Restricted to 234 endometriosis and 473 comparison participants who reported having periods in the last 3 months. Due to the phrasing in the questionnaire, participants who reported having periods could have been on cyclic hormone therapy or having bleeding despite being on continuous hormones.

^3^ P-value for any difference between three dysmenorrhea severity groups.

^4^ P-value for test of interaction for those with and without endometriosis.

^5^ Restricted to participants who answered the baseline questionnaire from January 2014 onwards when this question was added and to participants who reported having periods in the last 3 months (163 endometriosis and 417 comparison participants).

^6^ Missing dysmenorrhea frequency: 2 endometriosis and 6 comparison participants.

^7^ P-value for linear test for trend modeling the exposure as ordinal.

^8^ Missing presence of acyclic pelvic pain: 5 endometriosis and 4 comparison participants.

^9^ Among participants who reported acyclic pelvic pain in the past three months.

^10^ Missing acyclic pelvic pain severity: 9 endometriosis and 6 comparison participants.

^11^ Missing acyclic pelvic pain frequency: 8 endometriosis and 1 comparison participants.

^12^ Among participants who had intercourse in the past 12 months (excluded 182 endometriosis and 17 comparison aged <18 years, 3 endometriosis and 32 comparison who declined to be asked dyspareunia questions, 57 endometriosis and 82 comparison who had never had intercourse, 7 endometriosis and 11 comparison had not had intercourse in the past 12 months, 1 endometriosis and 3 comparison were missing information on dyspareunia).

^13^ Among participants age ≥18 with dyspareunia in past 12 months.

^14^ Missing dyspareunia severity: 1 endometriosis and 1 comparison participants.

^15^ Based on 0-10 numeric rating scale categorized as none/mild (0-3), moderate (4-6), severe (7-10).

^16^ Missing severity of pain with bowel movements: 17 endometriosis and 6 comparison participants.

**Table 4.** Presence, severity, and frequency of dysmenorrhea, acyclic pelvic pain, and dyspareunia as well as severity of pain with bowel movements in relation to 8-OHdG levels among all A2A participants without endometriosis (N=606) and A2A endometriosis participants who completed the baseline questionnaire and collected urine sample before their baseline surgery (N=313) ^1^.

	All participants		Endometriosis		No endometriosis	
		Creatinine-corrected 8-OhdG (ng/mg)		Creatinine-corrected 8-OhdG (ng/mg)		Creatinine-corrected 8-OhdG (ng/mg)
	N=919	Geometric mean (95% CI)	N=313	Geometric mean (95% CI)	N=606	Geometric mean (95% CI)
**Dysmenorrhea** ^**2**^						
Severity ^3^						
None/Mild	344 (49%)	80.3 (76.2, 84.6)	12 (6%)	82.0 (63.1, 106.6)	332 (65%)	81.6 (77.4, 86.1)
Moderate	190 (27%)	86.4 (80.6, 92.5)	57 (29%)	91.7 (81.3, 103.5)	133 (26%)	82.8 (76.1, 90.1)
Severe	170 (24%)	79.0 (73.3, 85.1)	126 (65%)	78.0 (72.0, 84.5)	44 (9%)	75.2 (64.9, 87.2)
*p-value* *^4^*		0.15		0.10		0.53
*p-interaction* *^5^*						0.66
Frequency ^6^ ^,^ ^7^						
Never/Rarely/Occasionally	307 (54%)	79.1 (74.8, 83.6)	13 (10%)	88.6 (67.5, 116.3)	294 (67%)	79.9 (75.5, 84.6)
Often/Usually	75 (13%)	87.7 (78.4, 98.2)	12 (9%)	99.9 (74.4, 134.1)	63 (14%)	85.7 (75.7, 97.0)
Always	187 (33%)	82.8 (77.0, 89.1)	104 (81%)	78.3 (71.2, 86.2)	83 (19%)	84.1 (75.5, 93.7)
*p-trend* *^8^*		0.27		0.21		0.32
*p-interaction* *^5^*						0.16
**Acyclic pelvic pain**						
Presence last 3 months ^9^						
No	623 (70%)	82.1 (79.1, 85.3)	111 (37%)	80.2 (73.7, 87.2)	513 (87%)	83.6 (80.1, 87.2)
Yes	267 (30%)	82.2 (77.5, 87.2)	189 (63%)	78.5 (73.7, 83.8)	78 (13%)	84.7 (75.9, 94.6)
*p-value*		0.97		0.71		0.82
*p-interaction* *^5^*						0.55
Severity ^10^ ^,^ ^11^						
Mild	42 (17%)	78.9 (68.5, 90.8)	17 (9%)	77.7 (63.5, 95.0)	25 (35%)	83.0 (66.9, 102.8)
Moderate	56 (22%)	80.5 (71.3, 90.9)	33 (18%)	77.5 (66.9, 89.6)	23 (32%)	85.8 (68.6, 107.4)
Severe	156 (61%)	81.2 (75.5, 87.3)	133 (73%)	80.7 (75.1, 86.7)	23 (32%)	79.9 (63.8, 100.0)
*p-trend* *^8^*		0.73		0.62		0.82
*p-interaction* *^5^*						0.49
Frequency ^10^ ^,^ ^12^						
Monthly or less	121 (46%)	82.2 (75.5, 89.5)	64 (35%)	82.1 (73.8, 91.4)	57 (74%)	86.0 (74.5, 99.3)
Weekly or daily	140 (54%)	78.7 (72.7, 85.1)	120 (65%)	77.5 (71.7, 83.8)	20 (26%)	75.9 (59.1, 97.5)
*p-value*		0.46		0.40		0.41
*p-interaction* *^4^*						0.63
**Dyspareunia** ^**13**^						
Presence in last 12 months						
No	329 (57%)	81.3 (77.1, 85.6)	25 (19%)	75.2 (64.0, 88.5)	304 (69%)	82.8 (78.3, 87.6)
Yes	246 (43%)	84.0 (79.1, 89.3)	110 (81%)	77.6 (71.9, 83.8)	136 (31%)	87.1 (80.0, 94.7)
*p-value*		0.42		0.74		0.34
*p-interaction* *^5^*						0.87
Severity ^14^ ^,^ ^15^						
Mild	82 (34%)	80.2 (72.6, 88.7)	25 (23%)	75.7 (64.2, 89.3)	57 (42%)	84.7 (74.1, 96.8)
Moderate	81 (33%)	85.2 (76.9, 94.3)	28 (26%)	78.4 (67.0, 91.7)	53 (39%)	89.4 (77.8, 102.6)
Severe	81 (33%)	80.9 (73.1, 89.5)	56 (51%)	76.5 (68.6, 85.4)	25 (19%)	84.9 (69.4, 103.9)
*p-trend* *^8^*		0.91		0.97		0.87
*p-interaction* *^5^*						0.86
Frequency ^14^						
Occasionally	117 (48%)	79.9 (73.5, 86.9)	27 (25%)	73.0 (62.5, 85.2)	90 (66%)	83.8 (75.6, 92.9)
Often/Usually	88 (36%)	87.3 (79.3, 96.1)	49 (45%)	79.9 (71.2, 89.6)	39 (29%)	95.8 (81.9, 112.0)
Always	41 (16%)	77.8 (67.5, 89.5)	34 (31%)	76.1 (66.2, 87.4)	7 (5%)	73.2 (50.3, 106.7)
*p-trend* *^8^*		0.87		0.74		0.61
*p-interaction* *^5^*						0.82
**Pain with bowel movements**						
Severity ^16^ ^,^ ^17^						
None/Mild	699 (79%)	82.0 (79.1, 85.0)	180 (61%)	78.8 (73.7, 84.3)	519 (88%)	83.3 (79.9, 87.0)
Moderate	112 (13%)	81.8 (74.8, 89.5)	61 (21%)	78.4 (69.9, 87.9)	51 (9%)	84.0 (73.3, 96.3)
Severe	72 (8%)	83.9 (74.9, 93.9)	54 (18%)	82.6 (73.1, 93.5)	18 (3%)	86.3 (68.6, 108.8)
*p-trend* *^8^*		0.76		0.57		0.77
*p-interaction* *^5^*						0.74

^1^ All p-values are two-sided and were adjusted for age (continuous in years), hormone use within the prior 30 days of urine collection (yes, no), pain medication use within the prior 48 hours of urine collection (yes, no), Alternative Healthy Eating Index (quartiles), body mass index (underweight, normal weight, overweight, obese), and physical activity (quartiles of MET-hrs/week). P-values for interactions between endometriosis and comparison participants were calculated using the Wald statistic.

^2^ Restricted to 197 endometriosis and 517 comparison participants who reported having periods in the last 3 months. Due to the phrasing in the questionnaire, participants who reported having periods could have been on cyclic hormone therapy or having bleeding despite being on continuous hormones.

^3^ Missing dysmenorrhea severity: 2 endometriosis and 8 comparison participants.

^4^ P-value for any difference between three dysmenorrhea severity groups.

^5^ P-value for test of interaction for those with and without endometriosis.

^6^ Restricted to participants who answered the baseline questionnaire from January 2014 onwards when this question was added and to participants who reported having periods in the last 3 months (132 endometriosis and 452 comparison participants).

^7^ Missing dysmenorrhea frequency: 3 endometriosis and 12 comparison participants.

^8^ P-value for linear test for trend modeling the exposure as ordinal.

^9^ Missing presence of acyclic pelvic pain: 13 endometriosis and 15 comparison participants.

^10^ Among participants who reported acyclic pelvic pain in the past three months.

^11^ Missing acyclic pelvic pain severity: 6 endometriosis and 7 comparison participants.

^12^ Missing acyclic pelvic pain frequency: 5 endometriosis and 1 comparison participants.

^13^ Among participants who had intercourse in the past 12 months (excluded 129 endometriosis and 17 comparison aged <18 years, 3 endometriosis and 36 comparison who declined to be asked dyspareunia questions, 35 endometriosis and 85 comparison who had never had intercourse, 6 endometriosis and 14 comparison had not had intercourse in the past 12 months, 5 endometriosis and 14 comparison were missing information on dyspareunia).

^14^ Among participants age ≥18 with dyspareunia in past 12 months.

^15^ Missing dyspareunia severity: 1 endometriosis and 1 comparison participants.

^16^ Based on 0-10 numeric rating scale categorized as none/mild (0-3), moderate (4-6), severe (7-10).

^17^ Missing severity of pain with bowel movements: 18 endometriosis and 18 comparison participants.

### Endometriosis lesion characteristics

Among the 380 endometriosis participants who had a WERF EPHect surgical form, the median time between surgery and urine collection was 13 days with an interquartile range of 0 days to 41 days. The vast majority of endometriosis participants had rASRM stage I/II disease (95%) and superficial peritoneal lesions only (96%; Table 5). There was a suggestion of lower 8-OHdG levels for participants with rASRM stage III/IV disease (GM=68.0; CI=55.4-83.5 ng/mg) compared to participants with rASRM stage I/II disease (GM=81.0; CI=77.3-84.9 ng/mg; p=0.10), although this was based on a small sample size of stage III/IV disease and thus limited power. These results remained similar when analyses were restricted to participants with urine collection prior to a baseline surgery ( Table 6).

**Table 5.** Endometriosis characteristics and creatinine-corrected 8-OHdG levels among A2A endometriosis participants ^1^ ^,^ ^2^.

	Endometriosis participants (n=434)		
		Creatinine-corrected 8-OHdG levels (ng/mg)	
	N	Geometric mean (95% CI)	p-value
Age at first endometriosis symptoms			
≤12 years	137 (32%)	81.8 (76.0, 88.1)	0.57
13 years	89 (21%)	80.1 (73.1, 87.7)	
14-15 years	113 (27%)	74.2 (68.4, 80.5)	
≥16 years	85 (20%)	80.8 (73.2, 89.1)	
Time between first symptoms and surgical diagnosis			
0 years	57 (13%)	77.5 (69.1, 87.0)	0.85
≤1 year	89 (21%)	86.1 (78.4, 94.5)	
>1-3 years	125 (29%)	74.9 (69.2, 81.1)	
>3 years	163 (38%)	79.2 (73.9, 84.9)	
Number of doctors seen for symptoms before diagnosis			
0-1	98 (26%)	81.7 (75.0, 89.1)	0.98
2-3	177 (47%)	79.6 (74.7, 84.8)	
4-5	63 (17%)	73.0 (65.6, 81.3)	
>5	35 (9%)	84.7 (73.3, 97.8)	
ASRM stage ^2^			
Stage I/II	344 (95%)	81.0 (77.3, 84.9)	0.10
Stage III/IV	19 (5%)	68.0 (55.4, 83.5)	
Endometriosis subtype ^2^			
Superficial peritoneal lesions only	361 (96%)	79.7 (76.0, 83.5)	0.36
Endometrioma	6 (2%)	75.9 (52.7, 109.3)	
Deep infiltrating	8 (2%)	112.5 (81.8, 154.9)	
Endometrioma and deep infiltrating	2 (1%)	56.2 (29.5, 107.0)	
**Endometriosis lesion color/vascularization** ^**3**^			
Any clear lesions			
No	25 (7%)	81.9 (67.9, 98.8)	0.76
Yes	355 (93%)	79.5 (75.8, 83.3)	
Any yellow lesions			
No	361 (95%)	78.9 (75.3, 82.7)	0.08
Yes	19 (5%)	95.3 (77.6, 116.9)	
Any red lesions			
No	61 (16%)	82.4 (73.1, 92.8)	0.55
Yes	319 (84%)	79.1 (75.2, 83.2)	
Any white lesions			
No	280 (74%)	78.5 (74.4, 82.8)	0.32
Yes	100 (26%)	82.8 (75.7, 90.6)	
Any blue/black lesions			
No	299 (79%)	81.6 (77.5, 85.9)	0.05
Yes	81 (21%)	72.8 (65.9, 80.5)	
Any brown lesions			
No	281 (74%)	81.5 (77.2, 85.9)	0.10
Yes	99 (26%)	74.6 (68.2, 81.7)	
Any clear, yellow or red lesions			
No	17 (4%)	76.6 (61.0, 96.1)	0.73
Yes	363 (96%)	79.8 (76.1, 83.6)	
Any white, blue/black or brown lesions			
No	181 (48%)	82.9 (77.6, 88.7)	0.10
Yes	199 (52%)	76.7 (72.0, 81.8)	
Any vascularized lesions			
No	263 (69%)	80.6 (76.3, 85.2)	0.44
Yes	117 (31%)	77.5 (71.3, 84.2)	
**Endometriosis lesion location** ^**3**^			
Any sidewall lesions			
No	108 (28%)	81.3 (74.6, 88.7)	0.57
Yes	272 (72%)	79.0 (74.8, 83.4)	
Any uterosacral ligament lesions			
No	333 (88%)	79.1 (75.3, 83.0)	0.41
Yes	47 (12%)	83.9 (73.5, 95.6)	
Any anterior cul-de-sac lesions			
No	180 (47%)	78.8 (73.7, 84.3)	0.68
Yes	200 (53%)	80.4 (75.4, 85.6)	
Any posterior cul-de-sac lesions			
No	37 (10%)	80.1 (68.8, 93.3)	0.94
Yes	343 (90%)	79.6 (75.8, 83.5)	
Any ovarian lesions			
No	367 (97%)	79.4 (75.8, 83.2)	0.58
Yes	13 (3%)	85.4 (66.2, 110.3)	
Any Fallopian tube/uterus lesions			
No	365 (96%)	79.8 (76.1, 83.6)	0.74
Yes	15 (4%)	76.5 (60.4, 96.9)	
Any bladder lesions			
No	377 (99%)	79.5 (76.0, 83.3)	0.61
Yes	3 (1%)	91.1 (54.0, 153.8)	
Any vagina lesions			
No	380 (100%)	79.6 (76.1, 83.4)	--
Yes	0 (0%)		
Any bowel lesions			
No	375 (99%)	79.6 (76.0, 83.3)	0.79
Yes	5 (1%)	84.0 (56.2, 125.5)	

^1^ Categories do not all add up to 434 cases due to missing values (age at first symptoms=10).

^2^ All p-values are two-sided and were adjusted for age (continuous in years), hormone use within the prior 30 days of urine collection (yes, no), pain medication use within the prior 48 hours of urine collection (yes, no), Alternative Healthy Eating Index (quartiles), body mass index (underweight, normal weight, overweight, obese), and physical activity (quartiles of MET-hrs/week).

^3^ Among endometriosis participants with a completed baseline WERF EPHect surgical form (N=380). Among the 380 endometriosis participants, 17 missing rASRM stage and 3 missing endometriosis subtype.

**Table 6.** Endometriosis characteristics and creatinine-corrected 8-OHdG levels among A2A endometriosis participants who collected urine samples before baseline surgery ^1^ ^,^ ^2^.

	Endometriosis participants (n=298)		
		Creatinine-corrected 8-OHdG levels (ng/mg)	
	N	Geometric mean (95% CI)	p-value
ASRM stage			
Stage I/II	281 (95%)	81.1 (77.0, 85.5)	0.10
Stage III/IV	14 (5%)	66.0 (51.9, 84.0)	
Endometriosis subtype			
Superficial peritoneal lesions only	289 (96%)	79.6 (75.5, 83.8)	0.41
Endometrioma	3 (1%)	69.0 (40.9, 116.5)	
Deep infiltrating	8 (3%)	111.8 (81.2, 154.0)	
Endometrioma and Deep infiltrating	2 (1%)	53.7 (28.1, 102.5)	
**Endometriosis lesion color/vascularization**			
Any clear lesions			
No	24 (8%)	82.3 (67.9, 99.8)	0.71
Yes	281 (92%)	79.2 (75.1, 83.6)	
Any yellow lesions			
No	287 (94%)	78.6 (74.6, 82.8)	0.09
Yes	18 (6%)	94.8 (76.8, 117.1)	
Any red lesions			
No	58 (19%)	84.3 (74.6, 95.3)	0.30
Yes	247 (81%)	78.4 (74.0, 83.0)	
Any white lesions			
No	216 (71%)	78.6 (73.9, 83.5)	0.51
Yes	89 (29%)	81.7 (74.2, 89.8)	
Any blue/black lesions			
No	235 (77%)	82.0 (77.3, 86.9)	0.03
Yes	70 (23%)	71.6 (64.3, 79.7)	
Any brown lesions			
No	225 (74%)	80.9 (76.2, 85.9)	0.26
Yes	80 (26%)	75.6 (68.4, 83.6)	
Any clear, yellow or red lesions			
No	16 (5%)	77.5 (61.2, 98.1)	0.83
Yes	289 (95%)	79.6 (75.5, 83.9)	
Any white, blue/black or brown lesions			
No	135 (44%)	83.8 (77.6, 90.6)	0.07
Yes	170 (56%)	76.2 (71.1, 81.6)	
Any vascularized lesions			
No	198 (65%)	80.7 (75.7, 86.1)	0.42
Yes	107 (35%)	77.2 (70.7, 84.3)	
**Endometriosis lesion location**			
Any sidewall lesions			
No	93 (30%)	82.4 (75.0, 90.5)	0.37
Yes	212 (70%)	78.2 (73.5, 83.2)	
Any uterosacral ligament lesions			
No	259 (85%)	78.6 (74.3, 83.1)	0.30
Yes	46 (15%)	84.8 (74.2, 96.8)	
Any anterior cul de sac lesions			
No	150 (49%)	78.3 (72.8, 84.3)	0.59
Yes	155 (51%)	80.6 (75.0, 86.6)	
Any posterior cul de sac lesions			
No	35 (11%)	79.6 (68.0, 93.2)	0.98
Yes	270 (89%)	79.5 (75.2, 83.9)	
Any ovarian lesions			
No	294 (96%)	79.3 (75.2, 83.5)	0.60
Yes	11 (4%)	85.4 (64.7, 112.9)	
Any Fallopian tube/uterus lesions			
No	292 (96%)	79.7 (75.6, 84.0)	0.67
Yes	13 (4%)	75.2 (58.2, 97.2)	
Any bladder lesions			
No	302 (99%)	79.4 (75.4, 83.6)	0.60
Yes	3 (1%)	91.5 (54.1, 154.8)	
Any vagina lesions			
No	305 (100%)	79.5 (75.5, 83.6)	
Yes	0 (0%)		
Any bowel lesions			
No	300 (98%)	79.4 (75.4, 83.6)	0.79
Yes	5 (2%)	83.9 (56.1, 125.4)	

^1^ Among endometriosis participants with a completed baseline WERF EPHect surgical form (N=298). Among the 298 endometriosis participants, 10 missing rASRM stage and 3 missing endometriosis subtype.

^2^ All p-values are two-sided and were adjusted for age (continuous in years), hormone use within the prior 30 days of urine collection (yes, no), pain medication use within the prior 48 hours of urine collection (yes, no), Alternative Healthy Eating Index (quartiles), body mass index (underweight, normal weight, overweight, obese), and physical activity (quartiles of MET-hrs/week).

For superficial peritoneal endometriotic lesion color, the highest 8-OHdG levels were observed for participants with any yellow lesions compared to those without (GM _yes_=95.3; CI=77.6-116.9 ng/mg vs. GM _no_=78.9; CI=75.3-82.7 ng/mg; p=0.08; Table 5). Additionally, endometriosis participants with white, blue/black or brown lesions had lower 8-OHdG levels compared to participants without (GM _yes_=76.7; CI=72.0-81.8 ng/mg vs. GM _no_=82.9; CI=77.6-88.7 ng/mg; p=0.10). This difference appeared to be driven by lower 8-OHdG levels among participants with any blue/black lesions compared to those without (GM _yes_=72.8; CI=65.9-80.5 ng/mg vs. GM _no_=81.6; CI=77.2-85.9 ng/mg; p=0.05) and similarly low levels among participants with brown lesions (p=0.10). Results for brown lesions were attenuated when analyses were restricted to participants with urine collection before their baseline surgery; however, results for having any yellow lesions, any blue/black lesions, and any white, blue/black or brown lesions remained similar ( Table 6). We did not observe any differences in 8-OHdG levels by lesion location.

## Discussion

In this cross-sectional analysis among a predominately adolescent and young adult population, we observed that pelvic pain symptoms were not associated with urinary 8-OHdG levels among either participants with or without endometriosis. Among endometriosis participants, rASRM stage III/IV disease was associated with lower levels of urinary 8-OHdG compared to participants with stage I/II disease; however this finding was based on a small number of stage III/IV disease participants. Further, lower 8-OHdG levels were observed for participants with white, blue/black, or brown lesions, suggesting that the role of oxidative stress in endometriosis pathophysiology may differ by lesion type.

### Pelvic pain

The majority of the previous studies on oxidative stress and pelvic pain symptoms have focused on dysmenorrhea with most observing an association between higher oxidative stress levels among those with dysmenorrhea. ^10^^–^^15^ The largest study to date with 897 adolescents observed that the serum pro-oxidant/antioxidant balance was shifted more towards the pro-oxidant side among participants with primary dysmenorrhea compared to those without dysmenorrhea when adjusting for age and BMI. ^10^ Conversely, Konishi *et al.* (2018) noted that severity of menstrual pain was not associated with urinary 8-OHdG levels among 188 female university students after adjusting for age and BMI. Similar to the results of Konishi *et al.* (2018), we observed that dysmenorrhea severity was not associated with 8-OHdG. Further, we observed that neither acyclic pelvic pain nor dyspareunia were associated with urinary 8-OHdG among participants with and without endometriosis. We may not have observed associations between 8-OHdG and pelvic pain symptoms in our study due to (1) assessing the DNA oxidative product of 8-OHdG given lipid or protein oxidation may have been more important for endometriosis-associated pelvic pain, (2) it may be that the interplay between oxidative stress and other molecules in the peritoneal cavity, such as inflammatory molecules, may be important for pelvic pain as opposed to oxidative stress on its own, or (3) the younger age of our study population (37% <21 years old) if associations between oxidative stress and pelvic pain are more apparent at older ages. Therefore, future research on other types of oxidative stress and the interplay between oxidative stress and the peritoneal microenvironment in relation to pelvic pain is needed.

### Endometriosis lesion characteristics

Oxidative stress has been implicated in the onset and progression of endometriosis and higher levels of oxidative products have been observed in the peritoneal fluid of individuals with endometriosis compared to control participants. ^29^ However, limited studies have assessed differences in oxidative stress levels among subsets of endometriosis patients and those that have, mainly focused on endometriosis patients presenting with infertility. In our study of mainly pain presenting endometriosis participants, we observed a suggestion of lower 8-OHdG levels for rASRM stage III/IV endometriosis compared to stage I/II; however, these results were based on a small sample of rASRM stage III/IV endometriosis participants. Contrary to our results, previous studies have observed increased oxidative stress with higher rASRM stage, ^11^^,^^14^^,^^30^^–^^32^ while two small studies among infertile endometriosis patients observed no association between endometriosis stage and lipid oxidation. ^33^^,^^34^ Differences between our results and the previous studies may be due to differences in the study populations with the younger, mostly pain presenting population in the A2A for which the association between oxidative stress and disease stage may be different from endometriosis patients who present with infertility. Additionally, differences between oxidative stress markers measured and biological sample types (e.g. blood, urine) utilized, as well as a lack of adjustment for potential confounders in the previous studies, may have led to differences between our results and previous studies. Finally, we noted that endometriosis participants with any white, blue/black or brown lesions had lower 8-OHdG levels compared to endometriosis participants with no white, blue/black or brown lesions, which appeared to be driven by the presence of blue/black lesions. To our knowledge, no other study has looked at lesion color and oxidative stress levels; therefore, these results warrant further exploration in other studies of endometriosis.

### Strengths and limitations

This study had some limitations including that we had only one marker of oxidative stress and thus may have missed associations between pelvic pain and protein and/or lipid oxidation. Additionally, some of the participants that have never been diagnosed with endometriosis within our study may have undiagnosed endometriosis; however, it is estimated that the community prevalence of undiagnosed endometriosis is <2%. Up to 10 years on since enrollment began in 2012, only four participants who at enrollment into our study had not been diagnosed with endometriosis, were subsequently diagnosed; the three participants who would have been eligible for these analyses were excluded. Effects of the characteristics of this small proportion of undiagnosed cases will be diluted among the true endometriosis-free participants. Further, as these analyses were cross-sectional, we cannot directly elucidate the cause and effect relationship between endometriosis lesion characteristics and 8-OHdG. Finally, the A2A population is predominately White, particularly among endometriosis participants; however, the population reflects the patients treated at the two participating hospitals and the general population of the people referred to those hospitals. Future research involving a more diverse population is needed.

Our study also had several strengths. It is one of the largest studies to date to assess oxidative stress and pelvic pain symptoms, and it included a predominately young population including endometriosis participants who are more proximal to their endometriosis symptom onset compared to previous studies. Although we only included one measure of DNA damage due to oxidative stress, urinary measurement of 8-OHdG has been validated as a reliable biomarker of oxidative stress in previous studies; and in comparison to blood samples, is less affected by potential misclassification due to oxidative processes that occur during sample collection and storage. Finally, we assessed multiple dimensions of pelvic pain, including presence, severity and frequency, which provided a more nuanced assessment of the relationship between pelvic pain symptoms and oxidative stress.

## Conclusions

Our results suggest that urinary 8-OHdG is not associated with dysmenorrhea, acyclic pelvic pain, dyspareunia or pain with bowel movements; however, 8-OHdG did appear to be differentially associated with endometriotic lesion color. Further research into differences in oxidative stress levels between endometriosis lesion types may help to further efforts to understand biologically and clinically informative subgroups of endometriosis patients, who may have different underlying biological processes and thus may respond differently to treatments. Investigations of additional oxidative stress markers among a large population of endometriosis patients with a focus on diversity in endometriosis subtypes will help to advance not only a greater understand of endometriosis pathophysiology but may also help in the development of novel therapeutics for pelvic pain symptoms.

## Data Availability

Data are not publicly available due to information that could compromise research participants’ privacy and consent. However, experienced scientists who would like to inquire regarding use of data from this study to address specific hypotheses or replicate the analyses in this study may submit an application and research proposal. Data requests must be reviewed and approved by the BWH Institutional Review Broad ( https://www.brighamandwomens.org/research/research-administration ). All inquiries should be directed to the A2A senior investigator and Boston Center for Endometriosis Scientific Director, Dr. Stacey Missmer ( smissmer@hsph.harvard.edu). Data sharing will require a fully executed Data Usage Agreement.
