# Supplementary material for: Pelvic pain symptoms and endometriosis characteristics in relation to oxidative stress among adolescents and adults with and without surgically-confirmed endometriosis
Source: F1000Res. 2024 Jan 8;13:34. [Version 1] doi: 10.12688/f1000research.141793.1 (PMC10940847; doi:10.12688/f1000research.141793.1)
Supplement: Peer review history

# Reviewer report: Reviewer response for version 1

**DOI:** 10.5256/f1000research.155270.r251912

**Published:** 2024-03-14

**Authors:** Renata Voltolini Velho

**Affiliations:** 1 Endometriosis Research Center Charité, Berlin, Germany

**Pelvic pain symptoms and endometriosis characteristics in relation to oxidative stress among adolescents and adults with and without surgically-confirmed endometriosis**

 Amy L Shafrir, Britani Wallace, Ashley Laliberte, Allison F Vitonis, Christine B Sieberg, Kathryn L Terry, Stacey A Missmer

 The study examined the relationship between pelvic pain symptoms and oxidative stress in individuals with and without surgically confirmed endometriosis. Although no links were found between 8-OHdG and pelvic pain symptoms, the research provides valuable insight into the variations in oxidative stress among endometriosis lesion sub-phenotypes. The paper boasts an impressive sample size and is well-structured and written. However, the study's major limitation is that it only used one marker of oxidative stress, which may have resulted in missed associations. Is there any possibility to include other oxidative stress markers?

Is the work clearly and accurately presented and does it cite the current literature?

Yes

If applicable, is the statistical analysis and its interpretation appropriate?

Yes

Are all the source data underlying the results available to ensure full reproducibility?

Yes

Is the study design appropriate and is the work technically sound?

Yes

Are the conclusions drawn adequately supported by the results?

Yes

Are sufficient details of methods and analysis provided to allow replication by others?

Yes

Reviewer Expertise:

Endometriosis, human genetics

I confirm that I have read this submission and believe that I have an appropriate level of expertise to confirm that it is of an acceptable scientific standard.

# Reviewer report: Reviewer response for version 1

**DOI:** 10.5256/f1000research.155270.r243123

**Published:** 2024-03-11

**Authors:** Sylvia Mechsner

**Affiliations:** 1 Endometriosis Research Center Charité, Berlin, Germany

The study investigated the correlation between pelvic pain symptoms and oxidative stress in individuals with and without surgically confirmed endometriosis. Although the research did not find any connections between 8-OHdG and pelvic pain symptoms, it offers valuable insight into the variations in oxidative stress among endometriosis lesion sub-phenotypes. The paper has an impressive sample size and is well structured and written. The use of only one marker for oxidative stress is a major limitation and may have resulted in missed associations. I have read this submission. I believe that I have an appropriate level of expertise to confirm that it is of an acceptable scientific standard.

Is the work clearly and accurately presented and does it cite the current literature?

Yes

If applicable, is the statistical analysis and its interpretation appropriate?

Yes

Are all the source data underlying the results available to ensure full reproducibility?

Yes

Is the study design appropriate and is the work technically sound?

Yes

Are the conclusions drawn adequately supported by the results?

Yes

Are sufficient details of methods and analysis provided to allow replication by others?

Yes

Reviewer Expertise:

Gynecology, Endometriosis

I confirm that I have read this submission and believe that I have an appropriate level of expertise to confirm that it is of an acceptable scientific standard.
